# Prevalence of Overweight and Obesity and Its Associated Factors among Preschool Children in Sub-Saharan Africa: a Systematic Review and Meta-analysis

**DOI:** 10.1016/j.advnut.2026.100594

**Published:** 2026-01-14

**Authors:** Abdu Hailu Shibeshi, Zeytu Gashaw Asfaw, Aragaw Asfaw Hasen, Kassaye Getaneh Arge, Nuru Mohammed Hussen, Abubeker Alebachew Seid, Abdulkerim Hassen Moloro, Hiwot Altaye Asebe, Etsay Woldu Anbesu, Dejen Kahsay Asgedom, Molla Getie Mehari, Bizunesh Fantahun Kase

**Affiliations:** 1 Department of Statistics, College of Natural and Computational Sciences, Samara University, Samara, Ethiopia; 2 Department of Epidemiology and Biostatistics, School of Public Health, Addis Ababa University, Addis Ababa, Ethiopia; 3 Department of Nursing, College of Medicine and Health Sciences, Samara University, Samara, Ethiopia; 4 Department of Public Health, College of Health Sciences, Mattu University, Mattu, Ethiopia; 5 Department of Medical Laboratory Science, College of Medicine and Health Sciences, Injibara University, Injibara, Ethiopia

**Keywords:** overweight/obesity, preschool children, sub-Saharan Africa, systematic review, meta-analysis

## Abstract

Overweight and obesity among preschool children have become significant public health concerns in sub-Saharan Africa (SSA), driven by rapid urbanization, economic growth, and dietary shifts. This systematic review and meta-analysis assessed the prevalence of overweight and obesity and its associated factors among preschool children in SSA. A comprehensive search of peer-reviewed and gray literature was conducted up to 29 January, 2025, using databases such as MEDLINE, ScienceDirect, Research4Life, and *African Journals of Online*. The study followed Preferred Reporting Items for Systematic Review and Meta-Analysis guidelines. Due to significant heterogeneity (*I*^2^ = 99.4%, *P* < 0.001) identified through Cochran’s Q statistic, a random-effects model was used to estimate the pooled prevalence. Publication bias was assessed with funnel plots and Egger’s test. A total of 27 studies (*n* = 30,805) were included; 77.78% of studies showed a low risk of bias per the Joanna Briggs Institute appraisal tool. The pooled prevalence of overweight/obesity was 14.77% [95% confidence interval (CI): 11.94%, 17.60%]. Key factors associated with overweight/obesity included being aged 2–3 y [odds ratio (OR) = 2.65; 95% CI: 1.73, 4.05] and 48–60 mo (OR = 1.99; 95% CI: 1.33, 2.98), spending over 2 h on screen activities (OR = 3.33; 95% CI: 1.89, 5.84), consuming sweet foods (OR = 2.55; 95% CI: 1.86, 3.48), and having an overweight mother (OR = 3.72; 95% CI: 1.30, 10.65). Given the high prevalence, interventions should focus on promoting healthy diets, reducing screen time, encouraging physical activity, and supporting maternal health and nutrition. The review was registered in PROSPERO as CRD42024560996.

**Statement of significance**

This study provides the first comprehensive systematic review and meta-analysis on the pooled prevalence and associated factors of overweight/obesity among preschool children in sub-Saharan Africa, highlighting key risk factors such as child age, maternal BMI, screen time, and dietary habits. Unlike previous studies that primarily relied on Demographic and Health Survey datasets, this research incorporates a broader range of data sources, offering a more representative estimation of the region’s growing childhood obesity burden.

## Introduction

Overweight and obesity among preschool children have emerged as significant public health challenges globally, with sub-Saharan Africa (SSA) experiencing a growing burden of this dual malnutrition phenomenon. Although undernutrition has historically been the primary focus in SSA due to its high prevalence, recent evidence indicates a rising trend in childhood overweight and obesity, particularly in urban areas and among higher socioeconomic groups [1,2]. This shift is attributed to rapid urbanization, dietary transitions toward energy-dense and processed foods, and reduced physical activity levels [3]. Rapid urbanization and economic growth in SSA drive a “nutrition transition” from traditional diets to energy-dense, processed foods and sugar-sweetened beverages (SSBs), heavily marketed and increasingly available. Concurrently, urban environments promote sedentary behaviors through reduced physical activity, increased screen time, and safety concerns limiting outdoor play. These shifts collectively create an obesogenic environment that heightens the risk of childhood obesity [[3], [4], [5]].

The coexistence of undernutrition and overweight/obesity within the same populations, households, and even individuals a phenomenon termed the “double burden of malnutrition” poses a complex challenge for healthcare systems in SSA, which are already strained by infectious diseases and nutritional deficiencies [6]. This dual burden can manifest as children who are stunted (impaired growth from chronic undernutrition) yet also carry excess adiposity, increasing their susceptibility to metabolic diseases.

Preschool children (aged 2–5 y) are particularly vulnerable to the adverse effects of overweight and obesity, as early-life weight gain is associated with an increased risk of noncommunicable diseases (NCDs) such as diabetes, cardiovascular diseases, and hypertension in adulthood [7]. Furthermore, childhood obesity can lead to psychosocial issues, including low self-esteem and poor academic performance, which may persist into later life [8]. Despite these risks, data on the prevalence and determinants of overweight and obesity among preschool children in SSA remain fragmented, with most studies focusing on school-aged children or adults [9,10].

The prevalence of overweight and obesity among preschool children in SSA varies significantly across regions, with higher rates observed in urban areas and among wealthier households [4]. This disparity is driven by factors such as increased consumption of energy-dense, nutrient-poor foods, reduced physical activity, and sedentary behaviors associated with urbanization and technological advancements [6]. Additionally, cultural perceptions that equate a higher body weight with good health and prosperity further exacerbate the problem [9]. Despite these trends, there remains a lack of comprehensive and up-to-date data on the prevalence, determinants, and regional variations of overweight and obesity among preschool children in SSA.

The consequences of childhood overweight and obesity extend beyond physical health, affecting psychosocial well-being and academic performance. Furthermore, the economic burden of managing obesity-related comorbidities places additional strain on already fragile healthcare systems in SSA [11]. Addressing this issue requires a nuanced understanding of the underlying factors contributing to overweight and obesity in this population, as well as context-specific interventions tailored to the unique socioeconomic and cultural contexts of SSA.

Despite these significant risks, data on the prevalence and determinants of overweight and obesity among preschool children in SSA remain fragmented. Although the growing recognition of childhood overweight and obesity as a public health issue in SSA has led to increased research, most studies have primarily focused on school-aged children, adolescents, and adults [9,10]. This focus has created a significant evidence gap for the preschool demographic (aged 2–5 y), a critical window for early intervention. Existing systematic reviews in SSA often rely solely on Demographic and Health Survey (DHS) datasets [[12], [13], [14], [15]], which, although valuable for national trends, may not capture the full breadth of community-based studies or adequately explore the associated risk factors specific to early childhood. Furthermore, to our knowledge, no previous systematic review and meta-analysis has comprehensively synthesized evidence on both the prevalence and the full range of associated factors (e.g., dietary, behavioral, maternal, and socioeconomic) of overweight and obesity specifically among preschool children across SSA.

This study aims to fill this precise gap. We will conduct a systematic review and meta-analysis to synthesize all available published and unpublished data, moving beyond DHS-centric analyses to provide a more nuanced and representative estimation of the burden of overweight and obesity among preschool children in SSA. By consolidating this evidence, our study will provide a robust evidence base to inform policymakers, healthcare providers, and public health practitioners. The findings will be critical for designing and implementing effective, context-specific interventions and policies tailored to the unique socioeconomic and cultural contexts of SSA to combat this emerging health challenge.

### Review questions

The following review questions provide a framework for this systematic review and meta-analysis:

1) What is the pooled prevalence of overweight and obesity among preschool children in SSA, and how does it vary across different regions?
2) What are the key risk factors associated with overweight and obesity among preschool children in SSA?

## Methods

### Reporting and protocol registration

The results of this systematic review and meta-analysis were prepared and reported using the PRISMA guideline [16]. The protocol has been registered in the PROSPERO database with a registration number of CRD42024560996.

### Study design, settings, information sources, and search strategies

This study was employed a systematic review and meta-analysis design to synthesize existing evidence on the prevalence and determinants of overweight and obesity among preschool children (aged 2–5 y) in SSA. The study was focused on the SSA region, encompassing all 48 countries within the region as defined by the United Nations. Studies conducted in both urban and rural settings were included to capture the diverse socioeconomic and environmental contexts that influence childhood overweight and obesity. Data from community-based surveys, health facility records, and nationally representative studies were considered to ensure a comprehensive representation of the population.

A comprehensive and systematic search of peer-reviewed literature and gray literature was conducted up to 29 January, 2025. The search spanned multiple electronic databases, including MEDLINE (via PubMed), ScienceDirect, *African Journals of Online* (AJOL), Research4Life, and the Google Scholar search engine. To minimize publication bias, gray literature was sought from academic repositories (e.g., OpenGrey, ProQuest Dissertations and Theses), government health ministry websites, and reports from international organizations (e.g., WHO, UNICEF) relevant to SSA. The search strategy was designed using the Condition, Context, Population framework (*CoCoPop*) to ensure comprehensiveness and relevance. This framework structured our approach around 3 core components:

• *Condition (Co)*: overweight, obesity, BMI, adiposity, etc.
• *Context (Co)*: SSA, all SSA country names.
• *Population (Pop)*: preschool children, child preschool, toddlers, 2–5 y. These elements were combined using Boolean operators (AND, OR) to create focused search queries.

A sample search strategy for MEDLINE/PubMed is provided below: [“preschool child” (MeSH Terms) OR “child, preschool”(MeSH Terms) OR “preschool children”(Text Word) OR “toddler”(Text Word)] AND [“overweight”(MeSH Terms) OR “obesity”(MeSH Terms) OR “overweight”(Text Word) OR “obes∗”(Text Word) OR “body mass index”(MeSH Terms)] AND [“sub-Saharan Africa”(MeSH Terms) OR “sub-Saharan Africa”(Text Word) OR “Africa South of the Sahara”(Text Word) OR (List of all SSA country names)]. The complete MEDLINE search strategy, along with detailed search strategies for the other databases, is provided in Supplemental File 1.

### Measurement outcome variables

The primary outcome variable for this study is the pooled prevalence of overweight and obesity among preschool children (aged 2–5 y) in SSA, measured using BMI-for-age *z*-scores based on the WHO growth standards. Overweight is defined as a BMI-for-age *z*-score >+2 SD, whereas obesity is defined as >+3 SD. Alternative measures, such as weight-for-height z-scores or percentiles, were used if BMI data are unavailable.

Secondary outcomes include determinants and risk factors such as socioeconomic status (e.g., household income, maternal education), dietary patterns (e.g., consumption of energy-dense foods, breastfeeding duration), physical activity levels, and environmental factors (e.g., urban compared with rural residence). Additionally, the study was examined regional and subgroup variations in prevalence across geographic areas, socioeconomic groups, and sex, as well as associated health outcomes like hypertension, insulin resistance, and psychosocial issues.

### Eligibility criteria

Studies were included based on the following criteria:

• *Study design*: observational studies (cross-sectional, cohort) and nationally representative surveys (e.g., DHS) were included. Reviews, commentaries, editorial letters, case reports, qualitative studies, and conference abstracts were excluded.
• *Participants*: studies involving apparently healthy preschool children aged 2–5 y.
• *Condition*: studies that reported the prevalence of overweight and/or obesity based on WHO growth standards (BMI-for-age *z*-score >+2 SD for overweight, >+3 SD for obesity) or a clearly defined alternative metric.
• *Context*: studies conducted in any SSA country.
• *Outcome*: studies that provided sufficient data to calculate prevalence and/or measure of association [e.g., odds ratios (OR)] for associated factors.
• *Methodological rigor*: only studies that clearly defined their measurement tools for exposure and outcome and reported a sample size calculation or included a sufficient sample (e.g., >100 participants) were considered to ensure methodological quality.

### Search guide

The “*CoCoPop* search guide” framework was employed to design a systematic and comprehensive search strategy, ensuring alignment with the study’s objectives. The framework focused on 3 core components: *Outcome/condition*, which targeted overweight/obesity; *population*, encompassing preschool children; and *context/setting*, restricted to SSA. By structuring the search strategy around these elements, the guide facilitated the identification of relevant studies, ensuring consistency and precision in capturing data specific to the epidemiology of overweight/obesity among preschool children in SSA. This approach was integral to the conception and execution of the search strategies in the study.

### Selection of studies

The Endnote X7 software reference manager was utilized to collect and organize search results, remove duplicate articles, and manage citations. The screening process consisted of 2 phases following an initial evaluation of article titles. In the first phase, the first 2 authors (AHS and ZGA) independently assessed titles and abstracts according to the predefined eligibility criteria. In the second phase, abstracts meeting the criteria were subjected to full-text screening. Only studies that received approval from both authors were included in the final review. Disagreements were resolved through discussion and consultation with the last author (BFK). Detailed reasons for exclusion were recorded for all studies not meeting the criteria, culminating in a finalized list of studies for data extraction.

### Data extraction and management

Two independent authors (AHS and BFK) extracted data from the eligible articles using a standardized template adapted from the Joanna Briggs Institute (JBI) data extraction form for systematic reviews and research syntheses. To ensure consistency and accuracy, the authors initially pilot-tested the data extraction process on a sample of articles using Microsoft Excel before applying it to the complete dataset.

The data extraction form included study characteristics such as the first author’s name, publication year, country, study design, sample size, response rate, quality rating, overweight/obesity measurement tool, prevalence of overweight/obesity, and risk of bias results. Any disagreements during the extraction process were resolved through active discussion to reach consensus, with a third author (AAH) consulted when necessary. This approach ensured the highest level of accuracy and consistency in capturing all essential information.

### Quality assessment of the studies

The methodological quality of included studies was critically appraised by 2 independent authors (AHS and BFK) using the standardized JBI critical appraisal checklists [17]. The appropriate checklist was applied based on study design: the 8-item checklist for analytical cross-sectional studies and the 11-item checklist for cohort studies [17]. Each item was scored as “Yes” (1 point), “No” (0 points), “Unclear” (0 points), or “Not Applicable” (excluded from total score). The total score was calculated as a percentage and used to categorize studies as having a low, moderate, or high risk of bias.

To ensure consistency and resolve discrepancies in appraisal, a structured process was followed:

• *Independent review*: the 2 authors initially assessed each study separately.
• *Consensus meeting*: authors met to compare scores and discuss any disagreements.
• *Adjudication*: if a consensus could not be reached, a third senior author (ZGA) was consulted to make the final decision. This multistep process ensured the objective and rigorous application of the quality assessment tool across all included studies.

### Data processing and statistical analysis

We utilized Microsoft Excel to extract the required data from the studies. Subsequently, these data were imported into the STATA version 17 statistical software for the purpose of analyzing the aggregated results. Measuring heterogeneity based on statistical findings, outcome presentations, and methodologies was done using the *I*^2^ statistic and a chi-squared test by Cochran’s Q statistic with a 5% significance level. *I*^2^ values of 25%, 50%, and 75% are considered indicative of low, moderate, and high heterogeneity, respectively [18]. Due to the anticipated clinical and methodological diversity among studies from different countries and settings, a random-effects meta-analysis model was deemed the most appropriate a priori. This model accounts for both within-study variance and between-study variance, providing a more conservative and generalizable estimate of the pooled effect size when true effect sizes are believed to vary across populations. According to the results of the statistical test, there was significant heterogeneity among the included original studies (*I*^2^ = 99.4%, *P <* 0.001); therefore, the random-effects meta-analysis model [19] was used to estimate the pooled prevalence.

To explore the potential sources of this heterogeneity, we preplanned subgroup analyses based on factors hypothesized to influence the prevalence of overweight/obesity: *1*) geographic region (to capture socioeconomic and cultural variations across SSA), *2*) country (to identify national-level disparities), and *3*) age group of children (as nutritional status and growth patterns vary significantly in early childhood). Metaregression was considered but deemed unsuitable due to the limited number of studies within each subgroup. The findings from these analyses are presented in Table 1.

**TABLE 1:** Results of subgroup analyses to explore sources of heterogeneity

Variables	Subgroup	Studies (*n*)	Sample size (*n*)	Prevalence (%) (95% CI)	*I*^2^ (%)	*P* value
Country						
	Ethiopia	7	8533	12.67 (9.20, 16.14)	93.5	≤0.001
	South Africa	5	2221	21.01 (11.14, 30.89)	97.9	≤0.001
	Nigeria	6	5343	17.20 (6.52, 27.89)	99.6	≤0.001
	Kenya	2	1763	21.71 (19.79, 23.63)	0.0	0.490
	Ghana	2	1085	5.99 (0.00, 13.17)^1^	91.2	≤0.001
	Malawi	1	4023	4.25 (3.63, 4.87)	0.0	≤0.001
	Cameroon	3	5436	12.93 (8.97, 16.89)	93.9	≤0.001
	Uganda	1	2401	5.00 (4.13, 5.87)	0.0	≤0.001
SSA regions						
	Eastern Africa	11	16,720	12.65 (9.23, 16.08)	98.3	≤0.001
	Western Africa	8	6428	14.42 (6.88, 21.95)	99.5	≤0.001
	Southern Africa	5	2221	21.01 (11.14, 30.89)	97.9	≤0.001
	Central Africa	3	5436	12.93 (8.97, 16.89)	93.9	≤0.001
Age of preschool children (y)						
	2–5	14	19,883	13.57 (9.48, 17.66)	99.2	≤0.001
	3–5	9	9094	15.52 (11.23, 19.81)	97.5	≤0.001
	4–6	1	884	2.60 (1.55, 3.65)	0.0	≤0.001
	At 3	1	162	45.68 (38.01, 53.35)	0.0	≤0.001
	3–6	2	782	13.48 (0.37, 26.60)	96.4	≤0.001

Abbreviations: CI, confidence interval; SSA, sub-Saharan Africa.

Statistical notes:

• Pooled prevalence estimates were computed using a random-effects meta-analysis (DerSimonian–Laird method).

• Heterogeneity was assessed using the *I*^2^ statistic: 0%–40% = low; 30%–60% = moderate; 50%–90% = substantial; 75%–100% = considerable.

• *P* values for heterogeneity are displayed only for subgroups with ≥2 studies; subgroups with a single study are indicated by “—.”

• CIs were calculated using the Wilson score method, appropriate for binomial proportions.

Subgroup definitions:

• Eastern Africa: Ethiopia, Kenya, Malawi, Uganda.

• Western Africa: Ghana, Nigeria.

• Southern Africa: South Africa.

• Central Africa: Cameroon.

Age-group classification: defined according to the age categories reported in the original studies; some studies contributed estimates to >1 age group.

1 Negative CI limit truncated to 0.00% as prevalence cannot be negative. The original estimate of –1.19% resulted from statistical modeling of small sample sizes with high variance; the true prevalence is interpreted as 0%–13.17%.

Publication bias was assessed visually with a funnel plot and statistically using Egger’s regression test, with a *P* value of <0.05 indicating potential bias. For associated factors, pooled OR with 95% confidence intervals (CIs) were calculated, and a *P* value of <0.05 was considered statistically significant. The quality of evidence was evaluated using the Grading of Recommendations, Assessment, Development, and Evaluations approach (GRADE) [20]. Findings are presented in text, tables, and figures.

## Results

### Search results

**FIGURE 1:**
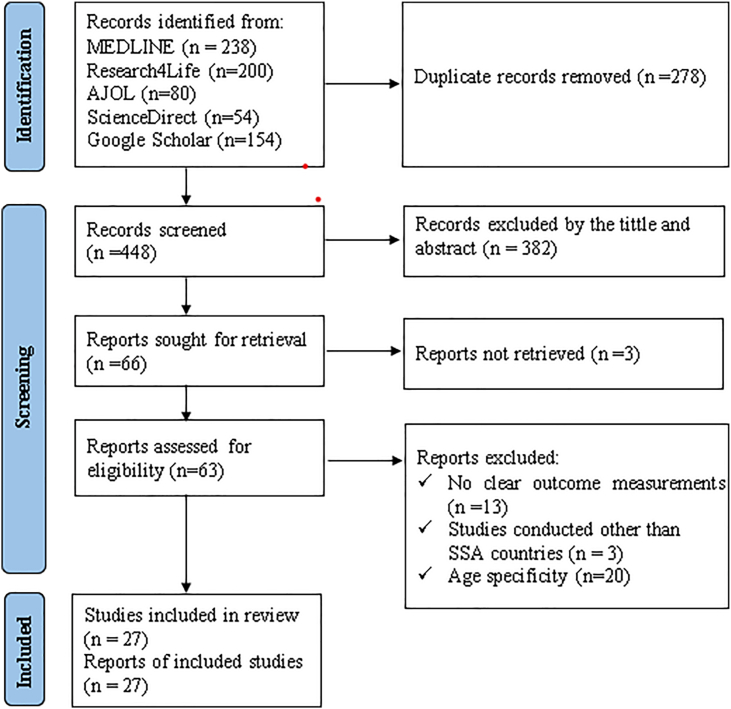
PRISMA 2020 flow diagram of the study identification and selection process, 2025. The figure details the number of records identified, screened, assessed for eligibility, and ultimately included in the systematic review and meta-analysis on overweight and obesity in preschool children in SSA. AJOL, *African Journals of Online*; SSA, sub-Saharan Africa.

A total of 726 studies were initially identified through various electronic databases, including MEDLINE, Research4Life, AJOL, ScienceDirect, and Google Scholar search engine. After removing 278 duplicate studies, 382 were excluded based on titles and abstracts that were unrelated to the research topic, and 3 additional articles were omitted due to inaccessible full reports. Subsequently, 63 full-text articles were assessed for eligibility based on preset criteria, resulting in the exclusion of 36 studies. Ultimately, 27 studies were included in this review. The study selection process is detailed in Figure 1, a PRISMA flow diagram that outlines the identification, screening, eligibility, and inclusion of the 27 studies analyzed in this review.

### Quality of included studies

The methodological quality of the 27 studies included in this review was assessed using the JBI quality assessment tool. Among them, 25 were cross-sectional studies [[21], [22], [23], [24], [25], [26], [27], [28], [29], [30], [31], [32], [33], [34], [35], [36], [37], [38], [39], [40], [41], [42], [43], [44], [45]], and 2 were cohort studies [46,47]. Studies were classified as high quality if they scored ≥6 for cross-sectional studies and ≥9 for cohort studies. As a result, 20 studies were rated as high quality, 6 as moderate, and 1 as low quality. All 27 studies, including the one that did not meet the high-quality criteria, were included in the final systematic review and meta-analysis (Table 2).

**TABLE 2:** Methodological quality assessment of included studies using the JBI critical appraisal tool, 2025

Included articles	Criteria (items 1–8 or 1–11)													
Author	Y	1	2	3	4	5	6	7	8	9	10	11	Total (%)	Overall quality
Berhane et al. [21]	2020	√	√	√	√	√	√	√	√	—	—	—	100	High
Ejara et al. [22]	2019	√	√	√	√	√	√	√	√	—	—	—	100	High
Faber et al. [23]	2001	√	√	√	√	ꭕ	ꭕ	√	ꭕ	—	—	—	62.5	Moderate
Folayan et al. [24]	2020	√	√	√	√	√	√	√	√	—	—	—	100	High
Folayan et al. [25]	2023	√	√	√	√	√	√	√	√	—	—	—	100	High
Gewa [26]	2010	√	√	√	√	√	√	√	√	—	—	—	100	High
Kumordzie et al. [46]	2020	√	√	√	√	ꭕ	√	√	ꭕ	√	√	ꭕ	72.7	Moderate
Mamabolo et al. [47]	2005	√	√	√	√	√	√	√	√	√	√	√	100	High
Manyong, et al. [27]	2021	√	√	√	√	√	√	√	ꭕ	—	—	—	87.5	High
Mezie-Okoye et al. [28]	2015	√	√	√	√	ꭕ	ꭕ	√	ꭕ	—	—	—	62.5	Moderate
Mokone et al. [29]	2022	√	√	ꭕ	√	ꭕ	ꭕ	√	ꭕ	—	—	—	50	Low
Motadi et al. [30]	2015	√	√	√	√	ꭕ	ꭕ	√	ꭕ	—	—	—	62.5	Moderate
Ntenda et al. [31]	2019	√	√	√	√	√	√	√	√	—	—	—	100	High
Odetunde et al. [32]	2014	√	√	√	√	ꭕ	ꭕ	√	ꭕ	—	—	—	62.5	Moderate
Okyere [33]	2018	√	√	√	√	√	√	√	√	—	—	—	100	High
Said-Mohamed et al. [34]	2009	√	√	√	√	√	√	√	√	—	—	—	100	High
Sebsbie et al. [35]	2022	√	√	√	√	√	√	√	√	—	—	—	100	High
Senbanjo et al. [36]	2007	√	√	√	√	ꭕ	ꭕ	√	ꭕ	—	—	—	62.5	Moderate
Senekal et al. [37]	2019	√	√	√	√	√	√	√	√	—	—	—	100	High
Sorrie et al. [38]	2017	√	√	√	√	√	√	√	√	—	—	—	100	High
Sserwanja et al. [39]	2021	√	√	√	√	√	√	√	√	—	—	—	100	High
Tadesse et al. [40]	2017	√	√	√	√	√	√	√	√	—	—	—	100	High
Tatah et al. [41]	2023	√	√	√	√	√	√	√	√	—	—	—	100	High
Tchoubi et al. [42]	2015	√	√	√	√	√	√	√	√	—	—	—	100	High
Tesedeke et al. [43]	2014	√	√	√	√	√	√	√	√	—	—	—	100	High
Wagaye et al. [44]	2024	√	√	√	√	√	√	√	√	—	—	—	100	High
Wandia et al. [45]	2014	√	√	√	√	ꭕ	ꭕ	√	√	—	—	—	75	High

Abbreviations: JBI, Joanna Briggs Institute; √, “Yes” (criterion fulfilled); X, “No” (criterion not fulfilled).

• JBI critical appraisal criteria for analytical cross-sectional studies (items 1–8): *1*) clear inclusion criteria; *2*) detailed description of subjects and setting; *3)* valid and reliable exposure measurement; *4*) objective criteria for condition measurement; *5*) confounding factors identified; *6*) strategies for confounding stated; *7*) valid and reliable outcome measurement; *8*) appropriate statistical analysis.

• Additional criteria for cohort studies (items 9–11): includes items 1–8 plus: *9*) groups comparable other than disease status; *10*) adequate follow-up time reported; *11*) complete follow-up or reasons for loss described.

• Quality rating classification: studies were rated as: high quality (low risk of bias): ≥6/8 criteria for cross-sectional studies (≥75%) or ≥9/11 criteria for cohort studies (≥82%); moderate quality: 5/8 criteria for cross-sectional studies (62.5%) or 7–8/11 criteria for cohort studies (64%–73%); low quality (high risk of bias): ≤4/8 criteria for cross-sectional studies (≤50%) or ≤6/11 criteria for cohort studies (≤55%).

• Two independent authors conducted quality assessments; discrepancies were resolved by consensus or third author consultation.

### Characteristics of the included studies

The final systematic review and meta-analysis included 25 cross-sectional studies and 2 cohort studies; all published between 2001 and 2024. A total of 27 articles were analyzed, with sample sizes ranging from 162 to 5822, encompassing 30,805 preschool children. Among the included studies, 7 were conducted in Ethiopia [21,22,35,38,40,43,44, 6[21,22,35,38,40,43,44], 6 in Nigeria [24,25,27,28,32,36, 5[24,25,27,28,32,36], 5 in South Africa [23,29,30,37,47, 3[23,29,30,37,47], 3 in Cameroon [34,41,42, 2[34,41,42], 2 in Ghana [33,46, 2[33,46], 2 in Kenya [26,45], and 2 in Malawi [31], and Uganda [39]. The response rates in the included primary studies varied from 86% in Kenya [26], to 100% in 20 other countries [21,[23], [24], [25],^28^,^29^,[31], [32], [33], [34], [35], [36], [37],^39^,[41], [42], [43], [44], [45],^47^] (Table 3).

**TABLE 3:** Characteristics of the 27 studies included in the systematic review and meta-analysis on overweight and obesity among preschool children in sub-Saharan Africa, 2025

Authors, year [ref]	Country	Survey year	Study design	Age range (y)	Sample size (*n*)	RR (%)	Overweight/obesity *n* (%)	Risk of bias^1^
Berhane et al., 2020 [21]	Ethiopia	2015–2016	CS	2–5	5822	100	664 (11.4)	Low
Ejara et al., 2019 [22]	Ethiopia	2016	CS	3–5	590	94.1	100 (16.9)	Low
Faber et al., 2001 [23]	South Africa	NR	CS	2–5	164	100	5 (3.0)	Moderate
Folayan et al., 2020 [24]	Nigeria	2018–2019	CS	3–5	1173	100	76 (6.5)	Low
Folayan et al., 2023 [25]	Nigeria	2019–2020	CS	3–5	1481	100	97 (6.5)	Low
Gewa, 2010 [26]	Kenya	2003 DHS	CS	3–5	1443	86	318 (22.0)	Low
Kumordzie et al., 2020 [46]	Ghana	2019	CoS	4–6	884	99.4	23 (2.6)	Moderate
Mamabolo et al., 2005 [47]	South Africa	NR	CoS	3^2^	162	100	74 (46.0)	Low
Manyong et al., 2021 [27]	Nigeria	2017	CS	2–5	1569	95.6	744 (47.4)	Low
Mezie-Okoye et al., 2015 [28]	Nigeria	2010	CS	2–5	220	100	52 (23.6)	Moderate
Mokone et al., 2022 [29]	South Africa	NR	CS	2–5	872	100	97 (11.1)	High
Motadi et al., 2015 [30]	South Africa	2013–2014	CS	3–5	349	92	107 (30.6)	Moderate
Ntenda et al., 2019 [31]	Malawi	2015–2016	CS	2–5	4023	100	171 (4.3)	Low
Odetunde et al., 2014 [32]	Nigeria	NR	CS	2–5	630	100	5 (0.8)	Moderate
Okyere 2018 [33]	Ghana	2018	CS	2–5	201	100	20 (9.9)	Low
Said-Mohamed et al., 2009 [34]	Cameroon	2008	CS	2–5	169	100	45 (26.6)	Low
Sebsbie et al., 2022 [35]	Ethiopia	2021	CS	2–5	224	100	13 (5.8)	Low
Senbanjo et al., 2007 [36]	Nigeria	NR	CS	2–5	270	100	51 (18.9)	Moderate
Senekal et al., 2019 [37]	South Africa	2018	CS	2–5	674	100	117 (17.3)	Low
Sorrie et al., 2017 [38]	Ethiopia	2016	CS	3–5	500	99.2	69 (13.8)	Low
Sserwanja et al., 2021 [39]	Uganda	2016 DHS	CS	2–5	2401	100	120 (5.0)	Low
Tadesse et al., 2017 [40]	Ethiopia	2015	CS	3–6	462	97	32 (6.9)	Low
Tatah et al., 2023 [41]	Cameroon	2018 DHS	CS	3–5	2623	100	284 (10.8)	Low
Tchoubi et al., 2015 [42]	Cameroon	2001 DHS	CS	2–5	2644	100	226 (8.5)	Low
Tesedeke et al., 2014 [43]	Ethiopia	2012	CS	3–5	358	100	38 (10.6)	Low
Wagaye et al., 2024 [44]	Ethiopia	2022	CS	3–5	577	100	138 (23.9)	Low
Wandia et al., 2014 [45]	Kenya	NR	CS	3–6	320	100	65 (20.3)	Low

Overweight/obesity prevalence reflects the proportion of preschool children with BMI-for-age > +2 SD according to WHO child growth standards.

Abbreviations: CoS, cohort study; CS, cross-sectional; DHS, Demographic and Health Survey; JBI, Joanna Briggs Institute; NR, not reported; ref, reference; RR, response rate.

1 Risk of bias assessment methodology: study quality was independently assessed by 2 authors using the JBI critical appraisal tools. For cross-sectional studies (*n* = 25), the 8-item JBI checklist was used. For cohort studies (*n* = 2), the 11-item JBI checklist was used. Discrepancies were resolved by consensus or third author consultation. Studies were classified as: low risk of bias (high quality): ≥6/8 criteria for cross-sectional studies or ≥9/11 for cohort studies; moderate risk: 5/8 criteria for cross-sectional studies or 7–8/11 for cohort studies; high risk of bias (low quality): ≤4/8 criteria for cross-sectional studies or ≤6/11 for cohort studies. Complete assessment criteria are detailed in Table 2.

2 Age at follow-up assessment for cohort study.

In terms of prevalence, the lowest (0.8%) and highest (47.4%) rates of overweight/obesity were reported in studies conducted in Nigeria [27,32]. The study with the lowest prevalence was conducted in Enugu Metropolis, a single urban area in southeastern Nigeria, whereas the study with the highest prevalence covered 5 states across different regions. The characteristics of the 27 included studies are summarized in Table 3, which provides key methodological and demographic details including sample size, country, study setting, response rate, year of publication, survey year and risk of bias assessment for each study.

### Risk of bias assessment

The risk of bias assessment of the included studies revealed that 20 studies had a low risk of bias, whereas 6 had a moderate risk, and 1 had a high risk of bias (Table 3).

### Result of publication bias assessment

**FIGURE 2:**
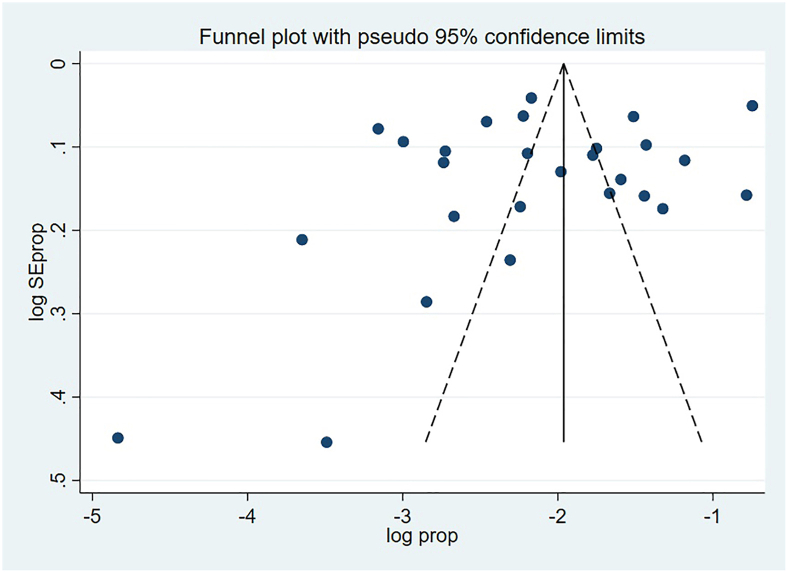
Funnel plot to assess potential publication bias for studies on the prevalence of overweight and obesity among preschool children in sub-Saharan Africa, 2025. The plot shows the SE of each study against its effect size (prevalence). The symmetrical distribution of studies around the pooled effect size (vertical line) suggests a low likelihood of publication bias.

Publication bias was assessed using a funnel plot and Egger’s regression test. Figure 2 presents the funnel plot, which shows a roughly symmetrical distribution of effect sizes around the pooled estimate, suggesting a low likelihood of publication bias. This visual interpretation was supported by a nonsignificant result from Egger’s test (*P* = 0.298). This suggests no evidence of publication bias among the studies.

### Sensitivity analysis test

**FIGURE 3:**
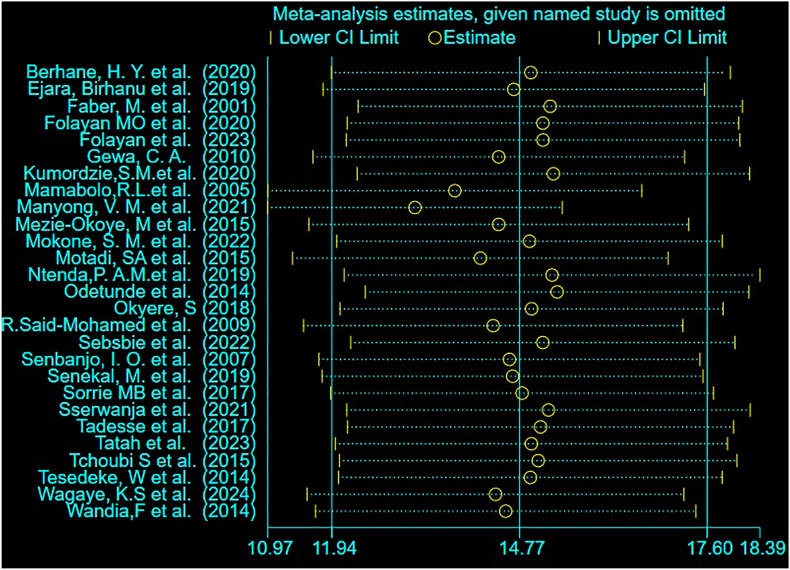
Leave-one-out sensitivity analysis of the pooled prevalence estimate of overweight and obesity among preschool children in sub-Saharan Africa, 2025. The plot shows the pooled prevalence and CI when each individual study is omitted sequentially. The stability of the overall estimate (solid vertical line) when any single study is removed confirms the robustness of the meta-analysis results. CI, confidence interval.

The leave-one-out sensitivity analysis revealed that no individual study significantly influenced the pooled prevalence of overweight/obesity among preschool children in SSA, confirming the robustness of the results (Figure 3).

### The result of estimated pooled prevalence of overweight and obesity among preschool children in SSA

**FIGURE 4:**
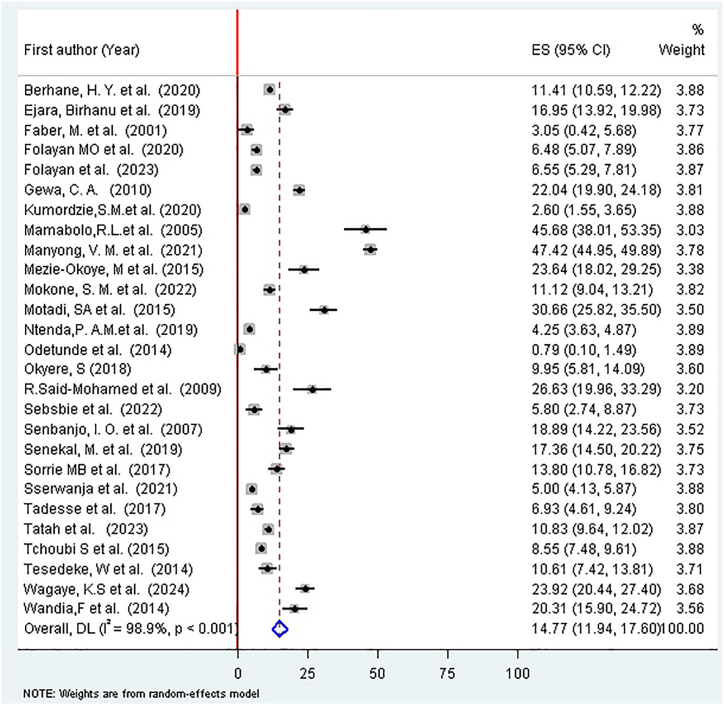
Forest plot of the pooled prevalence of overweight and obesity among preschool children in sub-Saharan Africa, 2025. The prevalence estimate for each individual study is represented by a square, with the size of the square proportional to the study’s weight in the analysis. The horizontal line through each square represents the study’s 95% CI. The diamond at the bottom represents the overall pooled prevalence estimate and its 95% CI, derived from a random-effects meta-analysis. CI, confidence interval.

A random-effects model was used to estimate the pooled prevalence of overweight and obesity among preschool children. The analysis indicated a pooled prevalence of 14.77% (95% CI: 11.94%, 17.60%) among preschool children in SSA, with substantial heterogeneity across the included studies (*I*^2^ = 98.9%, *P* < 0.001) (Figure 4). The 95% CI, while indicating a statistically significant estimate, is somewhat wide. This reflects the considerable variation in point estimates from the individual studies and underscores that the true prevalence across the diverse contexts of SSA likely lies within this range, rather than being a single precise value.

The considerable statistical heterogeneity observed (*I*^2^ = 98.9%, *P* < 0.001) indicates significant variation in the reported prevalence estimates beyond what would be expected by chance alone. This high heterogeneity is not unexpected given the substantial methodological and demographic diversity across the included studies, which encompassed multiple countries over a 24-y period with varying levels of urbanization, socioeconomic development, and cultural practices. It underscores that the pooled prevalence estimate should be interpreted as an summary measure across a wide spectrum of contexts within SSA rather than a single, uniform value. To investigate the potential sources of this heterogeneity, we conducted prespecified subgroup analyses.

### Subgroup analysis

To investigate the substantial heterogeneity and identify potential sources of variation, preplanned subgroup analyses were conducted based on factors hypothesized to influence the prevalence estimates: *1*) country and geographic region: To explore variations attributable to national and regional differences in economic development, food environments, and cultural norms, *2*) age group: to assess the influence of age-specific growth patterns and feeding practices.

The subgroup analyses revealed important variations. The pooled prevalence of overweight and obesity among preschool children was highest in Kenya (21.71%) and South Africa (21.01%), followed by Nigeria (17.2%), Cameroon (12.93%), and Ethiopia (12.67%). In contrast, the prevalence was lower in studies conducted in Ghana (5.99%), Uganda (5.00%), and Malawi (4.25%) (Table 3).

Regionally, the pooled prevalence was significantly higher in Southern Africa (21.01%) compared with other SSA regions (Western, Central, and Eastern Africa), suggesting regional socioeconomic factors play a key role. Furthermore, the prevalence was highest among 3-y-olds (45.68%), highlighting age as a critical determinant, potentially linked to specific feeding transitions during this period. The prevalence decreased in studies covering broader age ranges, including 3 to 5 y (15.52%), 2 to 5 y (13.57%), 3 to 6 y (13.48%), and 4 to 6 y (2.60%). Despite these explorations, significant residual heterogeneity remained within most subgroups (*I*^2^ > 90%, *P* < 0.001), indicating that other unmeasured factors, such as urban–rural disparities, socioeconomic status within countries, or differences in measurement techniques and year of study, also contribute to the observed variation (Table 3).

### Associated factors of overweight/obesity among preschool children in SSA

This systematic review and meta-analysis included 9 studies to examine the factors associated to overweight and obesity among preschool children in SSA [22,26,29, 31,37,38,[42], [43], [44]].

### Association between overweight/obesity and child age

**FIGURE 5:**
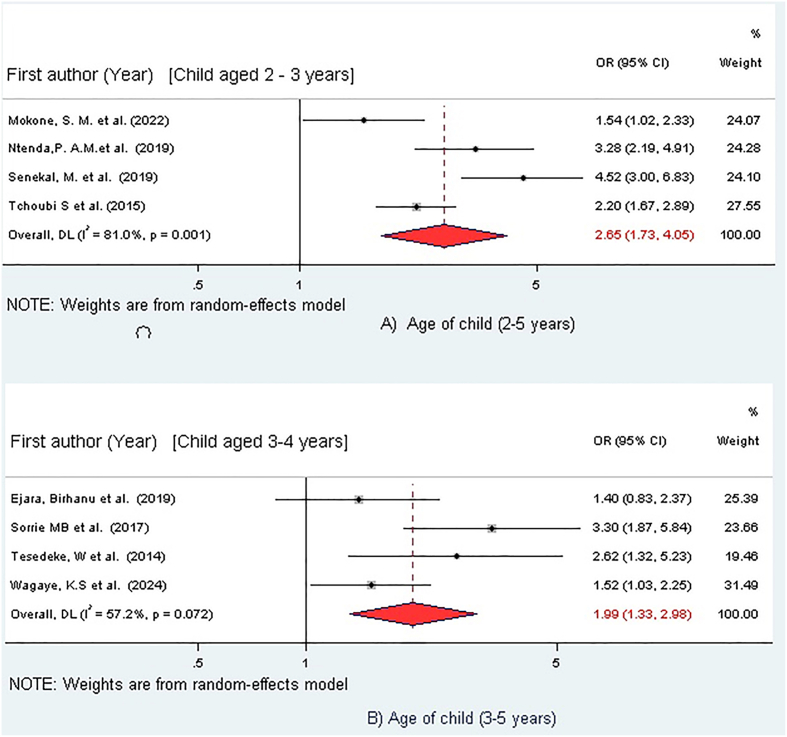
Association between child age and overweight/obesity among preschool children in sub-Saharan Africa, 2025. (A) Forest plot for the association between the 2–3 y age group (exposure) and overweight/obesity, using the 4–5 y group as the reference. (B) Forest plot for the association between the 36 and 47 mo age group (exposure) and overweight/obesity, using the 48–60 mo group as the reference. Each study is represented by a square (point estimate of the OR) and a horizontal line (95% CI). The size of the square corresponds to the study’s weight in the meta-analysis. The diamond represents the overall pooled OR and its 95% CI. The pooled analysis shows that younger children (2–3 y and 36–47 mo) had significantly higher odds of overweight/obesity compared with older peers. CI, confidence interval; OR, odds ratio.

Eight studies were included to examine the association between overweight/obesity and child age groups [22,29,31,37,38,[42], [43], [44]] (Figure 5). The age of the child (2–5 y) was significantly associated with overweight/obesity at the 5% significance level [29,31,37,42]. The findings revealed that children in the 2 to 3 y age group were 2.65 times more likely to be overweight/obese compared with those in the 4 to 5 y age group (OR =2.65, 95% CI: 1.73, 4.05) (Figure 5). Similarly, age in months (36–60 mo) was significantly associated with overweight/obesity [22,38,43,44]. Preschool children aged 36 to 47 mo had a 1.99 times higher risk of being overweight/obese compared with children aged 48 to 60 mo (OR = 1.99; 95% CI: 1.33, 2.98) (Figure 5).

### Association between overweight/obesity and time spent on watching TV/playing games and consuming sweet foods

**FIGURE 6:**
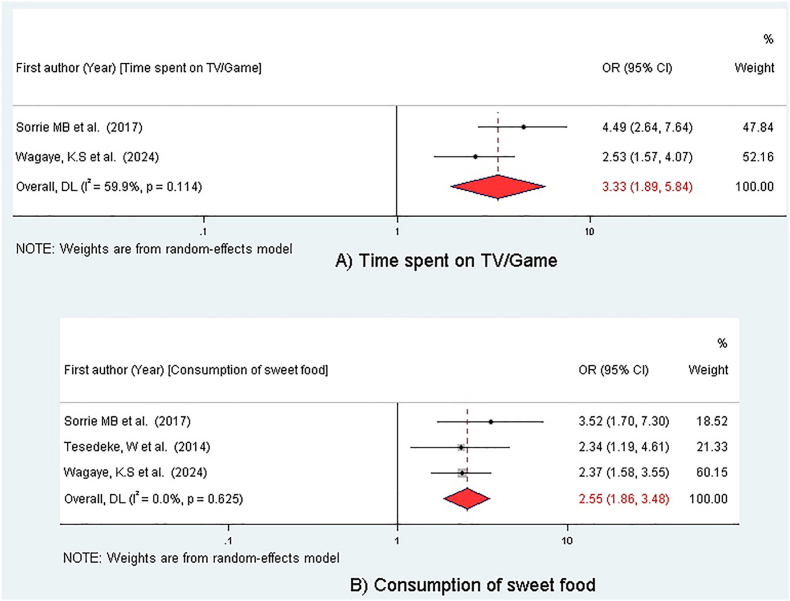
Association of behavioral factors with overweight/obesity among preschool children in sub-Saharan Africa, 2025. (A) Forest plot for the association between high screen time (>2 h/d) and overweight/obesity. (B) Forest plot for the association between sweet food consumption and overweight/obesity. For both plots, the reference group is children with low screen time (≤2 h/d) or no sweet food consumption, respectively. Each study is represented by a square (point estimate of the OR) and a horizontal line (95% CI). The size of the square corresponds to the study’s weight in the meta-analysis. The diamond represents the overall pooled OR and its 95% CI. The analysis shows that both high screen time and sweet food consumption are significantly associated with increased odds of overweight/obesity. CI, confidence interval; OR, odds ratio.

This meta-analysis included 3 studies [38,43,44], with 2 studies examining the association between overweight/obesity and time spent watching TV/playing games [38,44], and 3 studies investigating the association between overweight/obesity and the consumption of sweet foods [38,43,44]. Children who spent ≥2 h/d watching TV/videos or playing games had a 3.33 times higher risk of becoming overweight/obese compared with those who spent <2 h (OR = 3.33; 95% CI: 1.89, 5.84) (Figure 6).

In addition, the odds of being overweight/obese were 2.55 times higher for those children who consume sweet foods compared with those who don’t consume sweet foods (OR = 2.55; 95% CI: 1.86, 3.48) (Figure 6).

### Association between overweight/obesity and maternal nutritional status

**FIGURE 7:**
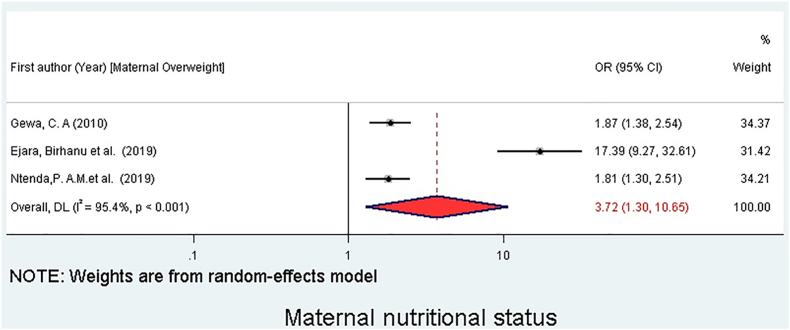
Association between maternal nutritional status and overweight/obesity in preschool children in sub-Saharan Africa, 2025. The forest plot shows the association between children having a mother with overweight or obesity (exposure) and the odds of the child being overweight or obese. The reference group is children of mothers with a normal weight or underweight nutritional status. Each study is represented by a square (point estimate of the OR) and a horizontal line (95% CI). The size of the square corresponds to the study’s weight in the meta-analysis. The diamond represents the overall pooled OR and its 95% CI. The pooled analysis indicates that children of mothers with overweight/obesity have significantly higher odds of being overweight/obese themselves. CI, confidence interval; OR, odds ratio.

Three studies were included to examine the association between overweight/obesity and maternal nutritional status [22,26,31]. Children from overweight and obese mothers were 3.72 times more likely to be overweight and obese as compared with children from nonoverweight and nonobese mothers (OR = 3.72; 95% CI: 1.30, 10.65) (Figure 7).

### Certainty of evidence using the GRADE approach

The certainty of evidence for prevalence is low due to high heterogeneity among studies, whereas the certainty for associated factors (child age, time spent on TV /game, sweet food consumption, and maternal BMI) is very low due to high risk of bias, imprecision, and moderate heterogeneity. This reflects the need for additional high-quality, geographically diverse studies using standardized diagnostic tools to address the observed heterogeneity and improve the precision of effect estimates. According to GRADE recommendations [48], further research is very likely to improve confidence in the effect estimates and may result in substantial changes to these estimates (Supplemental File 2).

## Discussion

This systematic review and meta-analysis were performed in SSA countries to determine the pooled prevalence of overweight/obesity and its associated factors among preschool children. This study will contribute to the development of public health strategies aimed at preventing and managing childhood overweight and obesity by promoting early detection, advocating for nutrition and lifestyle interventions, and supporting policies that foster a healthy environment for young children, ultimately improving their overall well-being in this population. Previous studies on the pooled prevalence of overweight and obesity among preschool children in SSA have primarily relied on DHS datasets, which were limited to a single-year DHS dataset for some countries [12,13,15]. In contrast, our study addresses this gap by incorporating a broader range of data sources for a more comprehensive and representative estimation.

This study included 27 studies with a total of 30,805 preschool children to estimate the pooled prevalence of overweight/obesity and its associated factors in SSA. Due to limited study availability in other countries, the analysis focused on research conducted in Ethiopia, Nigeria, South Africa, Cameroon, Ghana, Kenya, Malawi, and Uganda. Most included studies were cross-sectional, had small sample sizes, and were assessed as having a low risk of bias using the JBI checklist. The majority of primary studies came from Ethiopia (7), Nigeria (6), and South Africa (5), whereas Cameroon (3), Ghana (2), Kenya (2), Malawi (1), and Uganda (1) had fewer contributions. This limited geographic representation may affect the generalizability of the findings across the broader region. However, the study provides a valuable pooled prevalence estimate and highlights key factors associated with overweight/obesity among preschool children.

This meta-analysis estimated the pooled prevalence of overweight/obesity among preschool children in SSA at 14.77% (95% CI: 11.94%, 17.60%). The very high heterogeneity (*I*^2^ > 99%) observed across the studies underscores the substantial methodological and demographic diversity of the included research, suggesting that the pooled prevalence should be interpreted as an summary measure estimate across a wide range of contexts rather than a single, precise value. This heterogeneity was a key reason for employing a random-effects model and conducting extensive subgroup analyses. This prevalence is notably higher than that reported in 26 SSA countries using DHS datasets (6.8%) [12], African preschool children (8.5%) [14], Asia (4.9%) [14], Bangladesh among children and adolescents (7%) [49], and Iranian children and adolescents (10.8%) [50]. Additionally, it exceeds findings from studies conducted in specific SSA countries, including Zimbabwe (5.9%) and Malawi (4.5%) [15]. However, the prevalence observed in this study is lower than that reported among European preschool children (17.9%) [51], Iranian children under 5 y using a systematic review and meta-analysis (17%) [52], children in the Mediterranean region (25%) [53], and childhood overweight/obesity in Ghana (19.3%) [54].

The discrepancy in the prevalence of overweight/obesity among preschool children across different studies and regions can be attributed to several factors. One key reason is the differences in data sources and methodologies; this meta-analysis incorporated a wider range of studies beyond DHS datasets, which may have led to a higher pooled prevalence compared with previous estimates that relied solely on DHS data, typically focusing on national-level surveys with varying sampling methods. Socioeconomic factors and urbanization also play a role, as countries with higher rates of urbanization and economic development tend to have greater access to processed foods and sedentary lifestyles, which contribute to higher obesity rates [55,56]. This could explain why the prevalence in SSA is higher than in some developing regions like Asia and Bangladesh but lower than in European and Mediterranean countries.

Additionally, the dietary patterns and nutritional transitions in SSA, marked by a shift from traditional diets to energy-dense, processed foods, may be driving the rising rates of overweight/obesity, although this transition occurs at varying rates across regions [5,57]. Physical activity levels are another contributing factor, as differences in activity levels among children can influence obesity rates, with some SSA countries exhibiting lower obesity rates due to more active lifestyles, whereas higher rates in Europe and the Mediterranean may be linked to more sedentary behaviors [58]. Healthcare awareness and reporting differences also play a part; underreporting of childhood overweight/obesity in some low-income countries, due to limited health monitoring systems, may lead to lower prevalence estimates in DHS-based studies [59]. Cultural perceptions of body weight, where higher body weight may be associated with good health or wealth in certain SSA communities, can influence feeding practices, further contributing to higher obesity rates in specific countries [60]. Lastly, study selection bias may also explain some of the variation, as the studies included in this meta-analysis may have been conducted in regions with a higher burden of overweight/obesity, thus overestimating the overall prevalence compared with nationally representative DHS data [61,62].

There was significant heterogeneity in the prevalence of overweight/obesity among the studies included in this review. To identify potential sources of variation, a subgroup analysis was conducted. The findings revealed that Malawi (4.25%), Uganda (5%), and Ghana (5.99%) had the lowest pooled prevalence of overweight/obesity [31,33,39,46], whereas Kenya (21.71%) [26,45], and South Africa (21.01%) [23,29,30,37,47], reported the highest prevalence. The heterogeneity in overweight/obesity prevalence may be due to differences in urbanization and socioeconomic status, dietary patterns, healthcare awareness, cultural perceptions of body weight, and methodological variations between studies. Higher prevalence in Kenya and South Africa could be linked to greater urbanization, processed food consumption, and lifestyle changes, whereas lower rates in Malawi and Uganda may reflect rural lifestyles and traditional diets [63,64]. Additionally, differences in sample sizes across studies from these countries may have contributed to the observed variations in prevalence.

The subgroup analysis also revealed that preschool children aged 3 y had the highest prevalence (45.6%) in South Africa [47], whereas those aged 4 to 6 y had the lowest prevalence (2.6%) in Ghana [46]. The discrepancy may be due to differences in growth patterns, dietary habits, physical activity levels, socioeconomic factors, and sample size variations. Younger children (3 y) may experience rapid weight gain, have higher consumption of energy-dense foods, and engage in less physical activity, especially in urbanized settings like South Africa. In contrast, older children (4–6 y) tend to be more active, which may contribute to the lower prevalence observed in Ghana. Additionally, variations in sample sizes could influence the reported prevalence rates.

A key finding of this meta-analysis is the considerable heterogeneity (*I*^2^ > 98%) in the prevalence estimates across the included studies. This was explored through preplanned subgroup analyses by country, region, and age group. The significantly higher pooled prevalence observed in Southern Africa and Kenya aligns with their higher levels of urbanization and economic development, which are drivers of the nutrition transition toward energy-dense diets and more sedentary lifestyles [56,57]. The stark contrast in prevalence by age, with 3-y-olds being most affected, may reflect specific weaning practices and the early introduction of energy-dense complementary foods. However, the persistence of high heterogeneity within subgroups suggests that other important factors, such as urban versus rural setting, parental education, income inequality, and differences in study methodology over the long inclusion period, are influential. The use of a random-effects model was therefore essential, as it provides a more conservative and generalizable estimate that acknowledges this inherent diversity across SSA. Consequently, the pooled result represents a regional summary measure, and policymakers should interpret these findings within their specific national and subnational contexts.

The rising burden of childhood obesity in SSA presents a unique public health challenge, distinct from trends in high-income countries. Unlike elsewhere, this increase is often occurring within a context of persistent undernutrition and infectious diseases, creating a complex “double burden” that strains underresourced healthcare systems. This phenomenon is driven by a rapid and often uneven nutrition transition, characterized not by an overall surplus of food, but by a shift toward energy-dense, processed foods and sugary beverages amidst continued food insecurity. The implications are particularly acute in urban areas, where changing built environments, aggressive marketing of unhealthy commodities, and more sedentary lifestyles converge to create potent obesogenic environments. This necessitates a fundamental rethinking of public health strategies to integrate obesity prevention with existing efforts to combat undernutrition, moving from siloed programs to integrated approaches.

This review also examined factors associated with overweight/obesity among preschool children in SSA. Key factors identified included being in the 2 to 3 y age group, being within the 36 to 47 mo range, spending >2 h daily on TV or video games, consuming sweet foods, and having an overweight mother.

Our analysis showed that children aged 2 to 3 y, particularly those between 36 and 47 mo, had a higher prevalence of overweight and obesity. Children in this age group were more likely to be overweight or obese compared with those aged 49 to 60 mo. Similar findings in other studies suggest that younger preschool children face a greater risk of developing overweight and obesity [65]. A study in Cameroon also found that children <49 mo had higher odds of being overweight or obese than their older counterparts [42]. This trend may be linked to early-life nutritional practices and lower physical activity levels among younger children. Because diet and activity levels significantly influence weight, older preschoolers tend to be more active, leading to higher energy expenditure. However, some studies have not found a strong association between age and obesity, indicating that age-related vulnerability may differ across populations [66].

Spending >2 h watching TV or playing video games was identified as a factor associated with overweight/obesity in this study. This finding aligns with global evidence linking excessive screen time to childhood obesity [67]. Prolonged screen time encourages sedentary behavior, reduces physical activity, disrupts sleep patterns, and increases exposure to advertisements promoting unhealthy foods. Similar results have been reported in studies from Ethiopia [38,44], and Bosnia and Herzegovina [66]. This association may be due to reduced energy expenditure during screen time, a higher likelihood of consuming large food portions and sugary drinks, and the influence of food marketing on children’s eating habits. Additionally, disrupted sleep caused by excessive screen exposure may contribute to weight gain. However, some studies have not found a significant link between screen time and obesity in preschool children, suggesting that its impact may be influenced by other factors, such as the home environment and parental behaviors [68].

The consumption of sweet foods was significantly associated with overweight/obesity in this study. Children who consumed sweet foods were 2.55 times more likely to be overweight or obese compared with those who did not. Similar findings have been reported in studies from Hawassa, Ethiopia [43], and a systematic review in the Netherlands [69]. This association may be explained by the high acceptability of sweet foods among children, their energy-dense nature, and their potential to displace healthier food choices. Additionally, using sweet foods as a reward may encourage excessive consumption, whereas their affordability and widespread availability further contribute to increased intake.

Furthermore, our finding that consumption of sweet foods is a significant risk factor warrants a deeper discussion of the nutritional context in SSA. The term “sweet foods” in the included studies often encompasses a range of items central to the ongoing nutrition transition in the region, notably SSBs, commercially produced baked goods, candies, and sweetened cereals [5,57]. This shift away from traditional diets toward more processed, energy-dense, and often imported foods is particularly pronounced in urban areas and among higher socioeconomic groups, which aligns with our subgroup findings of higher prevalence in these settings [4,64]. The widespread availability and marketing of these palatable, low-nutrient foods, combined with increasing purchasing power, create an obesogenic environment that disproportionately affects young children. Therefore, the identified association with “sweet food consumption” likely reflects exposure to these broader unhealthy dietary patterns, highlighting a critical area for targeted policy interventions such as taxation on SSBs, regulation of junk food marketing to children, and programs promoting the value of traditional, nutrient-rich foods.

Maternal BMI emerged as a significant predictor of childhood overweight and obesity. This association may be attributed to genetic predisposition, shared dietary habits, and lifestyle behaviors between mothers and children. Overweight mothers are more likely to adopt feeding practices that promote excessive calorie intake, such as offering larger portion sizes or energy-dense foods, which can reinforce unhealthy eating patterns from an early age. Additionally, maternal perceptions of ideal body weight may influence child-feeding practices, with some mothers encouraging higher food consumption regardless of the child’s actual nutritional needs. Socioeconomic factors may also play a role, as families with overweight mothers may have greater access to processed and calorie-rich foods while engaging in lower levels of physical activity. This finding aligns with studies highlighting the impact of the home food environment and maternal BMI on children’s weight status [66,70,71].

Our findings point to several actionable strategies for policymakers. At the national level, we recommend implementing and enforcing policies that reduce children’s exposure to the marketing of unhealthy foods and SSBs, particularly on television and digital platforms. Fiscal policies, such as excise taxes (e.g., on SSBs), should be considered to deter consumption and generate revenue for child health programs. At the community and healthcare level, it is crucial to develop and promote simple, context-specific guidelines for healthy complementary feeding and screen time for young children. Furthermore, growth monitoring and counseling for overweight/obesity should be integrated into existing maternal and child health (MCH) programs to enable early detection. At the school and preschool level, establishing mandatory nutrition standards for meals and snacks provided in early childhood care and education centers is essential. These centers should also promote physical activity through play-based learning and ensure safe spaces for active play. These interventions should be tailored to regional realities; for example, high-prevalence regions in Southern Africa may require more stringent regulatory and fiscal measures, whereas in other regions, the initial focus might be on strengthening surveillance and integrating counseling into MCH services.

In conclusion, in this systematic review and meta-analysis, the pooled prevalence of overweight and obesity among preschool children in SSA was found to be significantly higher than that of children <5 in the region, with ∼1 in 7 preschool children affected. This review also offers valuable insights into the factors associated with overweight and obesity in preschool children. The findings revealed significant associations between overweight/obesity and factors such as being in the 2 to 3 y age group, being in the 36 to 47 mo range, spending >2 h watching TV or playing video games, consuming sweet foods, and having an overweight mother.

This high-prevalence signals a rapidly evolving public health challenge in SSA that demands a robust response. Although the policy implications for these modifiable risk factors are detailed in the discussion, this study also underscores critical evidence gaps that must be prioritized in future research. Specifically, there is a pressing need for longitudinal studies to establish causality, a greater focus on underrepresented regions such as Central Africa and rural settings, the standardization of measurement tools across studies, and implementation research to identify effective intervention delivery methods. Addressing the dual burden of malnutrition in SSA requires both the immediate implementation of evidence-based policies and a dedicated effort to generate higher-quality, context-specific research to guide those efforts effectively and avert a future surge in diet-related NCDs.

### Strengths and limitations of the study

This systematic review follows the PRISMA guidelines and provides a comprehensive analysis of the prevalence and associated factors of overweight and obesity among preschool children in SSA. To ensure quality and minimize bias, we conducted a thorough search across multiple databases and employed dual researcher screening. The review estimates pooled prevalence rates, identifies common associated factors, and performs subgroup analyses based on country, region, and preschool-age specifications. Researchers utilized the GRADE approach to assess the certainty of the evidence, ensuring reliable and transparent conclusions.

However, this study has several limitations. High heterogeneity among included studies and restricted geographic coverage limited to specific countries may affect the generalizability of the findings to other sub-Saharan nations. The focus on English-language publications may have excluded relevant research in other languages, and the scarcity of eligible studies from some countries further narrows the geographical scope. Additionally, while restricting the included population to a consistent preschool-age group improved comparability, it may have resulted in the omission of valuable insights. Some data were extracted from broader under-5 populations and aligned with preschool-age definitions specific to each setting, potentially leading to overestimation or underestimation of results. Imprecise estimates with wide CIs, often due to small sample sizes, further contribute to low certainty for prevalence and very low certainty for associated factors. Moreover, our findings should be interpreted considering our handling of missing data. The decision to exclude studies with critical missing numerical information, despite attempts to retrieve it from authors, was a conservative measure to ensure reliability but may have impacted the comprehensiveness of the pooled analysis. These findings highlight the need for more diverse, high-quality studies with standardized methodologies to strengthen the evidence base in this area.

## Author contributions

The authors’ responsibilities were as follows – AHS, ZGA, AAH, AAS, HAA, EWA, BFK: designed the research; AHS, ZGA, AAH, KGA, NMH, AAS, AHM, DKA, MGM, BFK: conducted abstract and full-text screening, data extraction, and risk o -bias assessment; AHS: developed the protocol, performed statistical analysis, and drafted the manuscript; AHS, ZGA, AAH, BFK: edited the manuscript; and all authors: reviewed and approved the final version, with AHS holding primary responsibility for the final content.

## Data availability

Data described in the manuscript, codebook, and analytic code will be made available upon request pending application and approval.

## Ethics approval

Not required.

## Consent for publication

Not applicable.

## Declaration of generative AI and AI-assisted technologies in the writing process

During the preparation of this work, the author(s) used QuillBot paraphrasing AI tool to enhance the manuscript’s clarity, readability, and overall quality by correcting grammatical errors and refining the language. After using this tool/service, the author(s) reviewed and edited the content as needed and take(s) full responsibility for the content of the publication.

## Funding

The authors reported no funding received for this study.

## Conflict of interest

The authors report no conflicts of interest.
