# Supplementary material for: Prevalence of Overweight and Obesity and Its Associated Factors among Preschool Children in Sub-Saharan Africa: a Systematic Review and Meta-analysis
Source: Adv Nutr. 2026 Jan 14;17(3):100594. doi: 10.1016/j.advnut.2026.100594 (PMC12908065; doi:10.1016/j.advnut.2026.100594)
Supplement: multimedia component 1 [file mmc1.docx]

**Overweight/obesity among preschool children in sub-Saharan Africa: A systematic review and meta-analysis**

**Search guide**

The **CoCoPop** search guide:

- **Condition:** Overweight/Obesity
- **Context**: Sub-Saharan Africa
- **Population:** Preschool children

**PubMed result (#238; January 29, 2025)**

| **Search Number** | **PubMed/MEDLINE Search detail** | **Result** |
| --- | --- | --- |
| **Condition/outcome: “Overweight/Obesity”** | | |
| 1 | ((((((((((((((((((((((((((((((((((((((((((((((((((((((((((((((((((((((((Overweight[MeSH Terms]) OR (Obesity[MeSH Terms])) OR ("Obesity Hypoventilation Syndrome"[MeSH Terms])) OR (Hypoventilation Syndrome, Obesity[Title/Abstract])) OR (Pickwickian Syndrome[Title/Abstract])) OR (Obesity-Hypoventilation Syndrome[Title/Abstract])) OR (Obesity-Hypoventilation Syndromes[Title/Abstract])) OR (Obesity, Abdominal[MeSH Terms])) OR (Abdominal Obesities[Title/Abstract])) OR (Obesities, Abdominal[Title/Abstract])) OR (Abdominal Obesity[Title/Abstract])) OR (Central Obesity[Title/Abstract])) OR (Central Obesities[Title/Abstract])) OR (Obesities, Central[Title/Abstract])) OR (Obesity, Central[Title/Abstract])) OR (Obesity, Visceral[Title/Abstract])) OR (Visceral Obesity[Title/Abstract])) OR (Obesities, Visceral[Title/Abstract])) OR (Visceral Obesities[Title/Abstract])) OR (Obesity, Metabolically Benign[MeSH Terms])) OR (Benign Obesity, Metabolically[Title/Abstract])) OR (Metabolically Benign Obesity[Title/Abstract])) OR (Metabolically Healthy Obesity[Title/Abstract])) OR (Healthy Obesity, Metabolically[Title/Abstract])) OR (Obesity, Metabolically Healthy[Title/Abstract])) OR (Obesity, Morbid[MeSH Terms])) OR (Morbid Obesities[Title/Abstract])) OR (Obesities, Morbid[Title/Abstract])) OR (Morbid Obesity[Title/Abstract])) OR (Obesity, Severe[Title/Abstract])) OR (Obesities, Severe[Title/Abstract])) OR (Severe Obesities[Title/Abstract])) OR (Severe Obesity[Title/Abstract])) OR (Pediatric Obesity[MeSH Terms])) OR (Obesity, Pediatric[Title/Abstract])) OR (Childhood Obesity[Title/Abstract])) OR (Obesity, Childhood[Title/Abstract])) OR (Obesity in Childhood[Title/Abstract])) OR (Child Obesity[Title/Abstract])) OR (Obesity, Child[Title/Abstract])) OR (Childhood Onset Obesity[Title/Abstract])) OR (Obesity, Childhood Onset[Title/Abstract])) OR (Adolescent Obesity[Title/Abstract])) OR (Obesity, Adolescent[Title/Abstract])) OR (Obesity in Adolescence[Title/Abstract])) OR (Childhood Overweight[Title/Abstract])) OR (Overweight, Childhood[Title/Abstract])) OR (Adolescent Overweight[Title/Abstract])) OR (Overweight, Adolescent[Title/Abstract])) OR (Infant Overweight[Title/Abstract])) OR (Overweight, Infant[Title/Abstract])) OR (Infantile Obesity[Title/Abstract])) OR (Obesity, Infantile[Title/Abstract])) OR (Infant Obesity[Title/Abstract])) OR (Obesity, Infant[Title/Abstract])) OR (Prader-Willi Syndrome[MeSH Terms])) OR (Prader Willi Syndrome[Title/Abstract])) OR (Syndrome, Prader-Willi[Title/Abstract])) OR (Labhart-Willi Syndrome[Title/Abstract])) OR (Labhart Willi Syndrome[Title/Abstract])) OR (Syndrome, Labhart-Willi[Title/Abstract])) OR (Labhart Willi Prader Fanconi Syndrome[Title/Abstract])) OR (Willi-Prader Syndrome[Title/Abstract])) OR (Syndrome, Willi-Prader[Title/Abstract])) OR (Willi Prader Syndrome[Title/Abstract])) OR (Prader Labhart Willi Syndrome[Title/Abstract])) OR (Prader-Labhart-Willi Syndrome[Title/Abstract])) OR (Syndrome, Prader-Labhart-Willi[Title/Abstract])) OR (Royer Syndrome[Title/Abstract])) OR (Syndrome, Royer[Title/Abstract])) OR (Royer's Syndrome[Title/Abstract])) OR (Royers Syndrome[Title/Abstract])) OR (Syndrome, Royer's[Title/Abstract]) | 337,206 |
| **Population: “Preschool children”** | | |
| 2 | (((Child, Preschool[MeSH Terms]) OR (Preschool Child[Title/Abstract])) OR (Children, Preschool[Title/Abstract])) OR (Preschool Children[Title/Abstract]) | 1,027,670 |
| **Context: “Sub-Saharan Africa”** | | |
| 3 | (((((((((((((((((((((((((((((((((((((((((((((((((((((((((((((((((((((((((((((((((((((((((((((((((((((((((((((((((((((((((((((((((((((((((((((Africa South of the Sahara[MeSH Terms]) OR (Sub-Saharan Africa[Title/Abstract])) OR (Subsaharan Africa[Title/Abstract])) OR (Africa, Sub-Saharan[Title/Abstract])) OR (Africa, Central[MeSH Terms])) OR (Central Africa[Title/Abstract])) OR (Cameroon[MeSH Terms])) OR (Republic of Cameron[Title/Abstract])) OR (United Republic of Cameroon[Title/Abstract])) OR (Cameroons[Title/Abstract])) OR (Central African Republic[MeSH Terms])) OR (Ubangi-Shari[Title/Abstract])) OR (Chad[MeSH Terms])) OR (Congo[MeSH Terms])) OR (Republic of the Congo[Title/Abstract])) OR (Congo (Brazzaville[Title/Abstract]))) OR (Democratic Republic of the Congo[MeSH Terms])) OR (Congo (Kinshasa[Title/Abstract]))) OR (Zaire[Title/Abstract])) OR (Belgian Congo[Title/Abstract])) OR (Katanga[Title/Abstract])) OR (Equatorial Guinea[MeSH Terms])) OR (Republic of Equatorial Guinea[Title/Abstract])) OR (Spanish Guinea[Title/Abstract])) OR (Guinea, Spanish[Title/Abstract])) OR (Rio Muni[Title/Abstract])) OR (Gabon[MeSH Terms])) OR (Gabonese Republic[Title/Abstract])) OR (Rwanda[MeSH Terms])) OR (Ruanda[Title/Abstract])) OR (Republic of Rwanda[Title/Abstract])) OR (Sao Tome and Principe[MeSH Terms])) OR (Africa, Eastern[MeSH Terms])) OR (East Africa[Title/Abstract])) OR (Eastern Africa[Title/Abstract])) OR (Burundi[MeSH Terms])) OR (Republic of Burundi[Title/Abstract])) OR (Urundi[Title/Abstract])) OR (Comoros[MeSH Terms])) OR (Iles Comores[Title/Abstract])) OR (Comoro Islands[Title/Abstract])) OR (Mayotte[Title/Abstract])) OR (Djibouti[MeSH Terms])) OR (Somaliland, French[Title/Abstract])) OR (Republic of Djibouti[Title/Abstract])) OR (French Somaliland[Title/Abstract])) OR (Eritrea[MeSH Terms])) OR (Ethiopia[MeSH Terms])) OR (Federal Democratic Republic of Ethiopia[Title/Abstract])) OR (Kenya[MeSH Terms])) OR (Republic of Kenya[Title/Abstract])) OR (Madagascar[MeSH Terms])) OR (Malagasy Republic[Title/Abstract])) OR (Seychelles[MeSH Terms])) OR (Somalia[MeSH Terms])) OR (South Sudan[MeSH Terms])) OR (Sudan[MeSH Terms])) OR (Republic of the Sudan[Title/Abstract])) OR (Tanzania[MeSH Terms])) OR (United Republic of Tanzania[Title/Abstract])) OR (Zanzibar[Title/Abstract])) OR (Tanganyika[Title/Abstract])) OR (Uganda[MeSH Terms])) OR (Republic of Uganda[Title/Abstract])) OR (Africa, Southern[MeSH Terms])) OR (Southern Africa[Title/Abstract])) OR (Angola[MeSH Terms])) OR (Botswana[MeSH Terms])) OR (Bechuanaland[Title/Abstract])) OR (Kalahari[Title/Abstract])) OR (Eswatini[MeSH Terms])) OR (Swaziland[Title/Abstract])) OR (Lesotho[MeSH Terms])) OR (Basutoland[Title/Abstract])) OR (Kingdom of Lesotho[Title/Abstract])) OR (Malawi[MeSH Terms])) OR (Republic of Malawi[Title/Abstract])) OR (Nyasaland[Title/Abstract])) OR (Mozambique[MeSH Terms])) OR (Republic of Mozambique[Title/Abstract])) OR (Portuguese East Africa[Title/Abstract])) OR (Namibia[MeSH Terms])) OR (Southwest Africa[Title/Abstract])) OR (Republic of Namibia[Title/Abstract])) OR (South West Africa[Title/Abstract])) OR (South Africa[MeSH Terms])) OR (Union of South Africa[Title/Abstract])) OR (Republic of South Africa[Title/Abstract])) OR (Zambia[MeSH Terms])) OR (Rhodesia, Northern[Title/Abstract])) OR (Northern Rhodesia[Title/Abstract])) OR (Republic of Zambia[Title/Abstract])) OR (Zimbabwe[MeSH Terms])) OR (Zimbabwe Rhodesia[Title/Abstract])) OR (Southern Rhodesia[Title/Abstract])) OR (Republic of Zimbabwe[Title/Abstract])) OR (Africa, Western[MeSH Terms])) OR (West Africa[Title/Abstract])) OR (Africa, West[Title/Abstract])) OR (Western Africa[Title/Abstract])) OR (Benin[MeSH Terms])) OR (Republic of Benin[Title/Abstract])) OR (Dahomey[Title/Abstract])) OR (Burkina Faso[MeSH Terms])) OR (Upper Volta[Title/Abstract])) OR (Burkina Fasso[Title/Abstract])) OR (Cabo Verde[MeSH Terms])) OR (Republic of Cape Verde[Title/Abstract])) OR (Cape Verde[Title/Abstract])) OR (Cote d'Ivoire[MeSH Terms])) OR (Ivory Coast[Title/Abstract])) OR (Republic of Cote d'Ivoire[Title/Abstract])) OR (Gambia[MeSH Terms])) OR (Republic of the Gambia[Title/Abstract])) OR (Ghana[MeSH Terms])) OR (Republic of Ghana[Title/Abstract])) OR (Gold Coast[Title/Abstract])) OR (Guinea[MeSH Terms])) OR (Guinea, French[Title/Abstract])) OR (Republic of Guinea[Title/Abstract])) OR (French Guinea[Title/Abstract])) OR (Guinea, Republic of[Title/Abstract])) OR (Guinea-Bissau[MeSH Terms])) OR (Republic of Guinea-Bissau[Title/Abstract])) OR (Portuguese Guinea[Title/Abstract])) OR (Guinea, Portuguese[Title/Abstract])) OR (Guinea-Bissau, Republic of[Title/Abstract])) OR (Liberia[MeSH Terms])) OR (Republic of Liberia[Title/Abstract])) OR (Mali[MeSH Terms])) OR (Republic of Mali[Title/Abstract])) OR (Mauritania[MeSH Terms])) OR (Niger[MeSH Terms])) OR (Republic of Niger[Title/Abstract])) OR (Nigeria[MeSH Terms])) OR (Federal Republic of Nigeria[Title/Abstract])) OR (Senegal[MeSH Terms])) OR (Republic of Senegal[Title/Abstract])) OR (Sierra Leone[MeSH Terms])) OR (Republic of Sierra Leone[Title/Abstract])) OR (Togo[MeSH Terms])) OR (Togolese Republic[Title/Abstract]) | 308,178 |
| 4 | ((#1) AND (#2)) AND (#3) | 238 |

**AJOL (#80)**

("preschool children" OR "children under five" OR "early childhood" OR "0–5 years" OR "toddlers" OR "infants") AND ("overweight" OR "obesity" OR "body mass index" OR "BMI" OR "adiposity" OR "excess weight" OR "childhood obesity") AND ("sub-Saharan Africa" OR "SSA" OR "Africa south of the Sahara" OR "Angola" OR "Benin" OR "Botswana" OR "Burkina Faso" OR "Burundi" OR "Cameroon" OR "Cape Verde" OR "Central African Republic" OR "Chad" OR "Comoros" OR "Congo" OR "Côte d'Ivoire" OR "Ivory Coast" OR "Democratic Republic of the Congo" OR "DRC" OR "Djibouti" OR "Equatorial Guinea" OR "Eritrea" OR "Eswatini" OR "Swaziland" OR "Ethiopia" OR "Gabon" OR "Gambia" OR "Ghana" OR "Guinea" OR "Guinea-Bissau" OR "Kenya" OR "Lesotho" OR "Liberia" OR "Madagascar" OR "Malawi" OR "Mali" OR "Mauritania" OR "Mauritius" OR "Mozambique" OR "Namibia" OR "Niger" OR "Nigeria" OR "Rwanda" OR "São Tomé and Príncipe" OR "Senegal" OR "Seychelles" OR "Sierra Leone" OR "Somalia" OR "South Africa" OR "South Sudan" OR "Sudan" OR "Tanzania" OR "Togo" OR "Uganda" OR "Zambia" OR "Zimbabwe")

**Research4Life (**using **HINARI** portal for health-related research**) (#200)**

("preschool children" OR "children under five" OR "early childhood") AND ("overweight" OR "obesity" OR "BMI") AND ("sub-Saharan Africa" OR "SSA" OR "Africa south of the Sahara" OR "Angola" OR "Benin" OR "Botswana" OR "Burkina Faso" OR "Burundi" OR "Cameroon" OR "Cape Verde" OR "Central African Republic" OR "Chad" OR "Comoros" OR "Congo" OR "Côte d'Ivoire" OR "Ivory Coast" OR "Democratic Republic of the Congo" OR "DRC" OR "Djibouti" OR "Equatorial Guinea" OR "Eritrea" OR "Eswatini" OR "Swaziland" OR "Ethiopia" OR "Gabon" OR "Gambia" OR "Ghana" OR "Guinea" OR "Guinea-Bissau" OR "Kenya" OR "Lesotho" OR "Liberia" OR "Madagascar" OR "Malawi" OR "Mali" OR "Mauritania" OR "Mauritius" OR "Mozambique" OR "Namibia" OR "Niger" OR "Nigeria" OR "Rwanda" OR "São Tomé and Príncipe" OR "Senegal" OR "Seychelles" OR "Sierra Leone" OR "Somalia" OR "South Africa" OR "South Sudan" OR "Sudan" OR "Tanzania" OR "Togo" OR "Uganda" OR "Zambia" OR "Zimbabwe")

**ScienceDirect (#54)**

(TITLE-ABS-KEY("preschool children") OR TITLE-ABS-KEY("children under five") OR TITLE-ABS-KEY("early childhood")) **AND** (TITLE-ABS-KEY("overweight") OR TITLE-ABS-KEY("obesity") OR TITLE-ABS-KEY("BMI")) **AND** (TITLE-ABS-KEY("sub-Saharan Africa") OR TITLE-ABS-KEY("SSA") OR TITLE-ABS-KEY("Africa south of the Sahara") OR TITLE-ABS-KEY("Angola") OR TITLE-ABS-KEY("Benin") OR TITLE-ABS-KEY("Botswana") OR TITLE-ABS-KEY("Burkina Faso") OR TITLE-ABS-KEY("Burundi") OR TITLE-ABS-KEY("Cameroon") OR TITLE-ABS-KEY("Cape Verde") OR TITLE-ABS-KEY("Central African Republic") OR TITLE-ABS-KEY("Chad") OR TITLE-ABS-KEY("Comoros") OR TITLE-ABS-KEY("Congo") OR TITLE-ABS-KEY("Côte d'Ivoire") OR TITLE-ABS-KEY("Ivory Coast") OR TITLE-ABS-KEY("Democratic Republic of the Congo") OR TITLE-ABS-KEY("DRC") OR TITLE-ABS-KEY("Djibouti") OR TITLE-ABS-KEY("Equatorial Guinea") OR TITLE-ABS-KEY("Eritrea") OR TITLE-ABS-KEY("Eswatini") OR TITLE-ABS-KEY("Swaziland") OR TITLE-ABS-KEY("Ethiopia") OR TITLE-ABS-KEY("Gabon") OR TITLE-ABS-KEY("Gambia") OR TITLE-ABS-KEY("Ghana") OR TITLE-ABS-KEY("Guinea") OR TITLE-ABS-KEY("Guinea-Bissau") OR TITLE-ABS-KEY("Kenya") OR TITLE-ABS-KEY("Lesotho") OR TITLE-ABS-KEY("Liberia") OR TITLE-ABS-KEY("Madagascar") OR TITLE-ABS-KEY("Malawi") OR TITLE-ABS-KEY("Mali") OR TITLE-ABS-KEY("Mauritania") OR TITLE-ABS-KEY("Mauritius") OR TITLE-ABS-KEY("Mozambique") OR TITLE-ABS-KEY("Namibia") OR TITLE-ABS-KEY("Niger") OR TITLE-ABS-KEY("Nigeria") OR TITLE-ABS-KEY("Rwanda") OR TITLE-ABS-KEY("São Tomé and Príncipe") OR TITLE-ABS-KEY("Senegal") OR TITLE-ABS-KEY("Seychelles") OR TITLE-ABS-KEY("Sierra Leone") OR TITLE-ABS-KEY("Somalia") OR TITLE-ABS-KEY("South Africa") OR TITLE-ABS-KEY("South Sudan") OR TITLE-ABS-KEY("Sudan") OR TITLE-ABS-KEY("Tanzania") OR TITLE-ABS-KEY("Togo") OR TITLE-ABS-KEY("Uganda") OR TITLE-ABS-KEY("Zambia") OR TITLE-ABS-KEY("Zimbabwe"))

**Google Scholar(#154)**

("preschool children" OR "children under five" OR "early childhood") AND ("overweight" OR "obesity" OR "BMI") AND ("sub-Saharan Africa" OR "SSA" OR "Africa south of the Sahara" OR "Angola" OR "Benin" OR "Botswana" OR "Burkina Faso" OR "Burundi" OR "Cameroon" OR "Cape Verde" OR "Central African Republic" OR "Chad" OR "Comoros" OR "Congo" OR "Côte d'Ivoire" OR "Ivory Coast" OR "Democratic Republic of the Congo" OR "DRC" OR "Djibouti" OR "Equatorial Guinea" OR "Eritrea" OR "Eswatini" OR "Swaziland" OR "Ethiopia" OR "Gabon" OR "Gambia" OR "Ghana" OR "Guinea" OR "Guinea-Bissau" OR "Kenya" OR "Lesotho" OR "Liberia" OR "Madagascar" OR "Malawi" OR "Mali" OR "Mauritania" OR "Mauritius" OR "Mozambique" OR "Namibia" OR "Niger" OR "Nigeria" OR "Rwanda" OR "São Tomé and Príncipe" OR "Senegal" OR "Seychelles" OR "Sierra Leone" OR "Somalia" OR "South Africa" OR "South Sudan" OR "Sudan" OR "Tanzania" OR "Togo" OR "Uganda" OR "Zambia" OR "Zimbabwe")
