# Supplementary material for: Prevalence of Overweight and Obesity and Its Associated Factors among Preschool Children in Sub-Saharan Africa: a Systematic Review and Meta-analysis
Source: Adv Nutr. 2026 Jan 14;17(3):100594. doi: 10.1016/j.advnut.2026.100594 (PMC12908065; doi:10.1016/j.advnut.2026.100594)
Supplement: multimedia component 2 [file mmc2.docx]

**Summary of the** **Certainty of Evidence using the GRADE Approach**

| **Outcome** | **Number of Studies** | **Study Design** | **Risk of Bias** | **Inconsistency** | **Indirectness** | **Imprecision** | **Publication Bias** | **Certainty of Evidence** |
| --- | --- | --- | --- | --- | --- | --- | --- | --- |
| Pooled prevalence of overweight/obesity | 27 | Cross-sectional & Cohort | Low to Moderate | High (I² = 98.9%) | No | Moderate | No significant bias (p = 0.298) | **Low** |
| Association with child age (2-5 years) and (3-5 years) | 8 | Cross-sectional | Moderate | High (I² = 81.0%) | No | Moderate | No assessment | Very Low |
| Association with time spent on TV/games | 2 | Cross-sectional | High | Moderate (I² = 59.92%) | No | High (p = 0.114) | No assessment | Very Low |
| Association with consumption of sweet foods | 3 | Cross-sectional | High | Moderate | No | High (p = 0.625) | No assessment | Very Low |
| Association with maternal nutritional status | 3 | Cross-sectional | High | Moderate | No | High (Wide CI: 1.30-10.65) | No assessment | **Very Low** |
